# Mechanical Properties of P91 Steel (X10CrMoVNb9-1) during Simulated Operation in a Hydrogen-Containing Environment

**DOI:** 10.3390/ma17174398

**Published:** 2024-09-06

**Authors:** Grzegorz Junak, Janusz Adamiec, Katarzyna Łyczkowska

**Affiliations:** Faculty of Materials Engineering, Silesian University of Technology, Krasińskiego 8, 40-019 Katowice, Poland; grzegorz.junak@polsl.pl (G.J.); janusz.adamiec@polsl.pl (J.A.)

**Keywords:** hydrogen permeation, hydrogen embrittlement, hydrogen induced cracking, P91 steel (X10CrMoVNb9-1/9Cr-1Mo-V)

## Abstract

P91 steel (X10CrMoVNb9-1) is widely used in the energy industry. It is characterized by good mechanical properties, creep resistance, corrosion resistance, impact toughness, and resistance to thermal fatigue. Due to their operating conditions and martensitic structure, components made from P91 steel are often subject to damage related to the presence of hydrogen. This article compares the results of the mechanical properties evaluation for P91 steel in an aggressive solution charged under load and without load. Based on the research, it was found that the hydrogen environment significantly affects the mechanical properties of P91 steel, reducing strength and yield strength, and decreasing ductility. It was revealed that in samples tested after 72 h without preloading, the tensile strength decreased by 1.5%, and the elongation decreased by about 29% for the sample, compared to the delivered condition sample. Under loaded conditions, the difference in tensile strength increased by approximately 8%, while elongation increased by nearly 50%

## 1. Introduction

**Figure 1:**
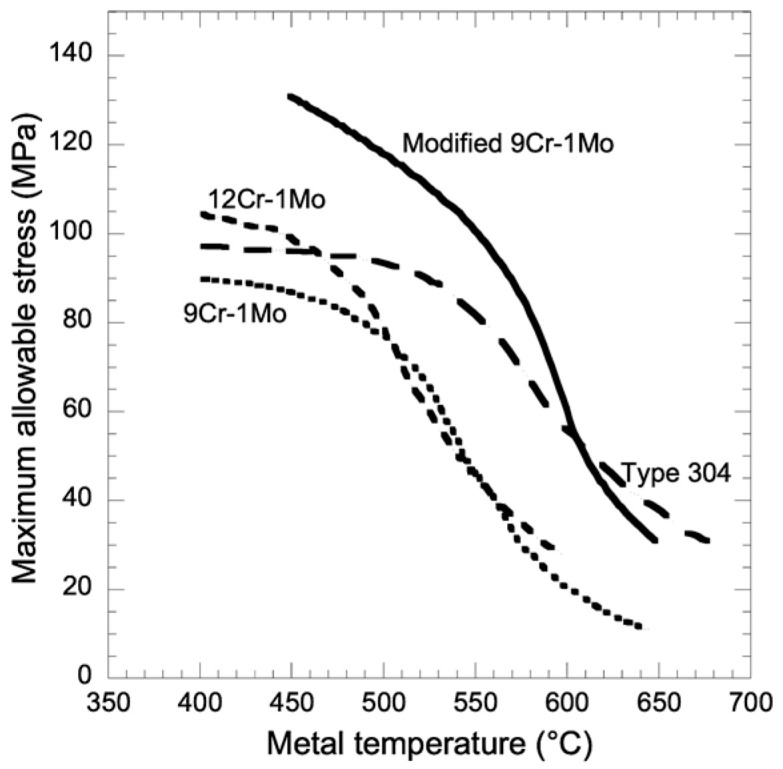
A comparison of maximum allowable stress as a function of temperature for P91 (modified 9Cr-1Mo), P22 (12Ce-1Mo), P9 (Fe-9Cr-1Mo), and 304 steel [8].

P91 steel (X10CrMoVNb9-1/9Cr-1Mo-V) is a martensitic steel with 9% Cr, 1% Mo, micro-additives of niobium and vanadium, and a controlled nitrogen content. It is used, e.g., in superheaters and preheater tubes in supercritical boilers [1,2,3]. The recommended maximum operating temperature is 650 °C [4,5,6,7]. The allowable temperature-dependent operating stresses calculated for P91 steel, compared to P22 (12Cr-1Mo steel), P9 (Fe-9Cr-1Mo), and 304 steel are shown in Figure 1. The modified steel P91 (addition of Nb, V, and N) has higher allowable stress intensities at temperature than the annealed P22 steel. P91 martensitic steel has worse properties than 304 austenitic steel, characterized by a maximum allowable stress below 50 Mpa, above a temperature of approximately 620 °C.

In the as-delivered condition, P91 steel is normalized at 1040–1080 °C, which results in the dissolution of carbides without significant grain growth. Then, at 750–780 °C, carbides precipitate uniformly throughout the martensite tempering process [1,9,10,11]. These are M23C6 carbide types rich in V/Nb. The microstructure also contains rich carbonitrides of the MX type (M = V or Cr; X = C or N). The presence of these precipitates significantly increases creep rupture strength through precipitation hardening. In the as-delivered condition, the strength of P91 steel is a minimum of 585 MPa, and the yield strength is no less than 415 MPa, with an A5 elongation of 20% [12,13,14,15,16].

Due to their operating conditions and martensitic structure, components made from P91 steel are also subject to damage related to the presence of hydrogen. Hydrogen damage is a very unfavorable phenomenon affecting many industries [17,18,19].

Factors determining the phenomena associated with the presence of hydrogen include hydrogen penetration into metals, hydrogen diffusion, hydrogen trapping, and stress and strain [20].

The presence of hydrogen in a material can cause hydrogen gas embrittlement, decreased ductility, internal cracking and delamination, segregation cracking, snowflakes and fish eyes, cold cracking, e.g., following a welding process [21,22,23], stress cracking accompanying the formation of hydrides, and the action of hydrogen during transformations, such as martensitic transformation, as well as SOHIC (Stress-Oriented Hydrogen-Induced Cracking) and HIC (Hydrogen-Induced Cracking) [24].

The literature also presents numerous mechanisms describing various phenomena related to the influence of hydrogen within materials. Among the commonly accepted mechanisms are three primary ones, which can act either independently or in concert. These are hydrogen-enhanced local plasticity (HELP), hydrogen-enhanced decohesion (HEDE), and adsorption-induced dislocation emission (AIDE) [25].

Hydrogen-enhanced local plasticity (HELP) is attributed to increased dislocation mobility in the presence of hydrogen. The accumulation of hydrogen reduces the dislocation stress field, thereby locally decreasing the dislocation cross-stress [26]. Consequently, a lower external stress is required to induce dislocation movement compared to an environment without hydrogen.

Another phenomenon contributing to embrittlement is hydrogen-enhanced decohesion (HEDE). HEDE describes the embrittlement of metals due to reduced bonding forces between matrix atoms caused by local accumulations of stored hydrogen. This local accumulation, often occurring at grain boundaries or phase interfaces, significantly lowers the energy required to initiate crack propagation [27].

In the case of adsorption-induced dislocation (AIDE), hydrogen embrittlement arises from a reduction in surface energy caused by hydrogen adsorption. The accumulation of hydrogen around precipitates or pores significantly reduces surface energy, thereby promoting microcrack formation. [28] Other descriptions of hydrogen-related mechanisms are provided in the work [15].

According to [19], the hydrogen damage process can be divided into hydrogen absorption, diffusion, and trapping, which eventually leads to cracking. There are several methods for evaluating a material’s resistance to hydrogen-induced damage: testing under constant stress on smooth or notched specimens, testing under constant strain, e.g., under constant tensile strain or at a given bending rate, and testing under slow load increase [24]. An important element in the determination of the hydrogen resistance of a material are corrosion tests: SSC (Sulphide Stress Cracking), according to NACE TM 0177-96, and HIC, according to NACE TM 208 [29,30,31].

However, SSC and HIC tests are not static tests, which means they do not provide an answer to the behavior of the material during simultaneous loading. An interesting test is the Slow Strain Rate Test (SSRT) in solution. There are numerous articles in the literature on SSRT tests conducted on materials dedicated to the energy industry, e.g., on hydrogen gas transportation pipelines [32,33,34,35] with an austenitic or ferritic–pearlitic structure, but there are definitely fewer studies on martensitic steels.

Interesting research on martensitic steels is described in the paper in [36]. The authors carried out the SSRT test with 3.5% NaCl, 1 g/L thiourea, and 1 mA/cm^2^ current density on two low-carbon martensitic steels Fe-0.068 C-0.39 Ti (Ti-containing steel) and Fe-0.069 C-0.45 Mo (Mo-containing steel). They found that the crack initiation in Mo-containing steel originated from Mo_2_C carbides, accompanied by decohesion at the interface between the martensitic matrix and Mo_2_C carbides, and that the hydrogen trapping capacity at the designated hydrogen trap sites was a determinant factor for H-assisted crack initiation and propagation in martensitic steels. They also found that the Ti-containing steel indicates a mitigated hydrogen embrittlement susceptibility, controlled by carbon vacancies inside TiC acting as strong hydrogen trap sites.

Other interesting research on the influence of the strain rate was presented in the article in [37]. According to this survey, the yield strength was not affected considerably by temperature (the experiment was conducted at 25 °C and 50 °C) and only affected by strain rate due to the effect on dislocation annihilation/motion, and the hydrogen embrittlement susceptibility increased with decreasing strain rate. They also found that more pronounced hydrogen embrittlement in low strain rates is due to the ability of hydrogen to interact with mobile dislocations, which leads to hydrogen-induced fracture.

Equally satisfactory research on the SSRT test with different current densities from 0.1 to 10 mA/cm^2^ was carried out on two martensitic steels with different Mo content (0.43 and 1.06 wt. %), which are presented in the paper in [38]. The authors showed that the crack growth rate increased and the values of stress intensity factors K_IH_ and K_Imax_ decreased with the increase in pre-charged hydrogen concentration. The steel exhibiting a higher molybdenum content demonstrated a significantly reduced crack growth rate compared to the steel with a lower Mo content. They showed it could be attributed to the presence of a greater density of nano-sized precipitates. These precipitates act as effective hydrogen trapping sites, effectively mitigating the detrimental effects of hydrogen on the crack propagation rate.

The only available study on the behavior of P91 steel during the SSRT test is described in [39], where Bharasi et al. conducted tests in a 1–4 M sodium hydroxide (NaOH) solution. The authors observed secondary stress corrosion cracks in all specimens tested in 1–4 M NaOH and the number of secondary cracks increased with an increase in concentration up to 3 M. Tensile test data showed that ductility decreased with an increasing concentration of NaOH up to 3 M, and practically remained the same for 4 M NaOH solution.

Despite numerous corrosion tests, the behavior of a material exposed to hydrogen is sometimes difficult to predict. Similarly, due to the complexity of factors affecting the acceleration or slowing down of structural degradation, it is very difficult to correctly estimate the effect of hydrogen on the durability of an industrial component or facility. In order to select the best possible test methodology, it is important to identify the exact operating conditions (including the operating pressure, temperature, and environment).

The results of available research show that there are few articles about the corrosion resistance of P91 steel at room temperature and elevated temperatures, however, there is very little information on the research around martensite P91 steel under the influence of hydrogen under load.

It therefore seems necessary to conduct research that will contribute to the description of the microstructure of P91 steel in the SSRT test. This paper compares the results of tests aimed at determining the mechanical properties of P91 steel following its charging with hydrogen, both under load and without load. The results unambiguously confirm a deterioration in the mechanical properties of P91 steel under the influence of hydrogen, in particular under load.

## 2. Materials and Methods

**Figure 2:**
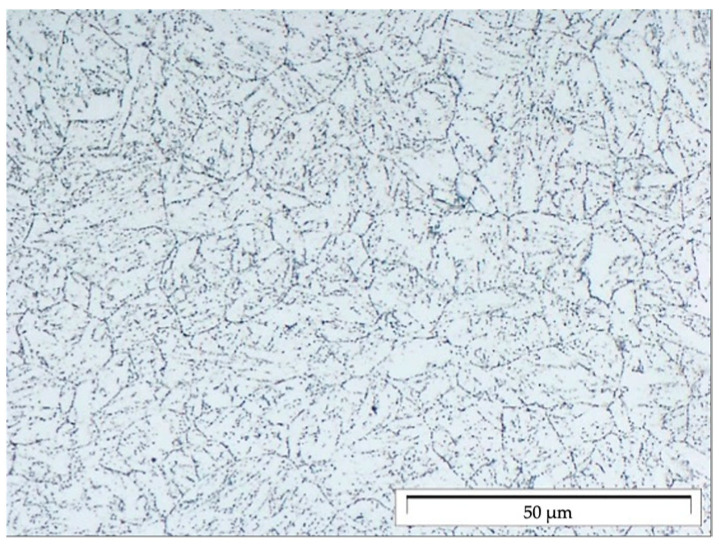
Martensitic structure of P91 steel in the as-delivered condition.

**Figure 3:**
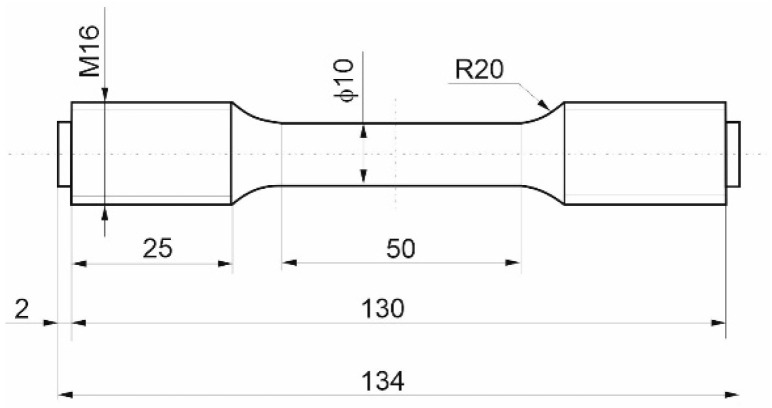
Specimen for testing the effect of hydrogen on the mechanical properties of P91 steel.

High-alloyed steel P91 (X10CrMoVNb9-1) with a martensitic structure designed for service at elevated temperatures was used for the tests. The structure in the as-delivered condition is shown in Figure 2. The tests were carried out on cylindrical specimens (in accordance with PN-EN ISO 6892-1) [40] with the geometry shown in Figure 3.

**Figure 4:**
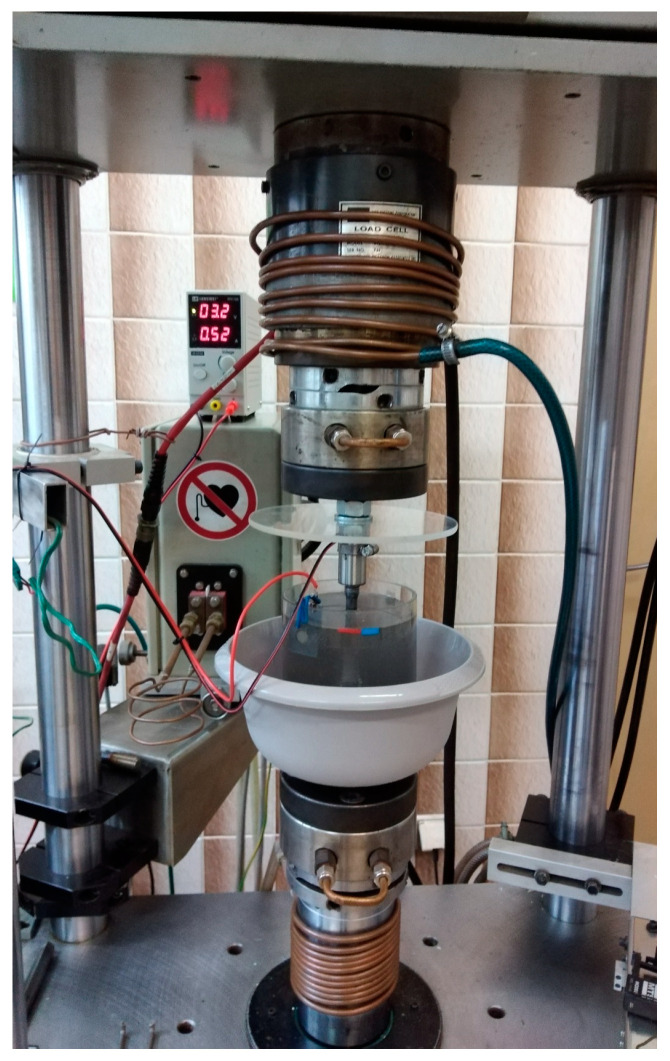
Stand for hydrogen charging under load.

The tests were carried out on a specially prepared stand (Figure 4) with the MTS-810 fatigue machine (250 kN) (MTS Systems, Eden Prairie, MN, USA). The stand allowed the hydrogen charging of the specimens while applying a tensile preload of a specified value. The tests were carried out at the Faculty of Materials Engineering of the Silesian University of Technology on a stand based on the MTS-810 250 kN fatigue machine. The stand enables electrolytic rehydration in a solution of 0.5 M H_2_SO_4_ and 25 mg/L As_2_O_3_. The current flow used in the tests was 10 mA/cm^2^. These conditions simulate the operation of pipelines in power installations and tanks for transporting hydrogen under high pressure.

In the first part, a static tensile test was carried out on the material in the as-delivered condition, in accordance with PN-EN ISO 6892-1, to determine the basic mechanical properties of the P91 steel tested. On this basis, the value of the preload to be applied during the hydrogen charging of other specimens was determined. The preload value selected was 70% of yield strength Rp_0.2_, which corresponded to approx. 380 MPa.

The remaining specimens (18) were electrolytically charged in 0.5 M H_2_O + 25 mg/L As_2_O_3_ solution. Half of the specimens (9) were charged under load, and the other half (9) without load. In both groups, 3 specimens were hydrogen-charged for 24 h, 3 specimens for 48 h, and 3 specimens for 72 h. The results presented below show the average for each category of specimens.

Following the hydrogen charging process, static tensile strength tests were carried out on the specimens under displacement control at a constant rate of 4 mm/min.

Throughout the tests, the following values were continuously recorded: time, displacement, force, and strain. An MTS-623.11c.20 extensometer (MTS Systems, Eden Prairie, MN, USA)with a gauge length of 25 mm was used to measure strain values.

Based on the results obtained, static tensile stress diagrams were developed, also including accurate measurements of material elongation following the hydrogen charging with and without load.

On this basis, the basic strength and plasticity properties of P91 steel were determined. Subsequently, the fracture surfaces and transverse and longitudinal cross-sections were subjected to metallographic examinations under a Hitachi S-3400N scanning microscope (Hitachi, Ltd., Tokyo, Japan) using the secondary electron detection technique. Images were recorded in the secondary electron mode and at a voltage accelerating the electron beam to 25 keV.

## 3. Results and Discussion

The study aimed to assess the mechanical properties and determine the structural phenomena that determine the hydrogen embrittlement of martensitic P91 steel (Figure 3, 14a) exposed to a strongly hydrogenating environment. Such conditions often occur in power installations, and, due to its high strength properties, it can be used in installations for transporting compressed hydrogen. For this reason, it is important to understand and confirm the mechanisms that affect the tendency toward hydrogen-assisted cracking.

**Figure 5:**
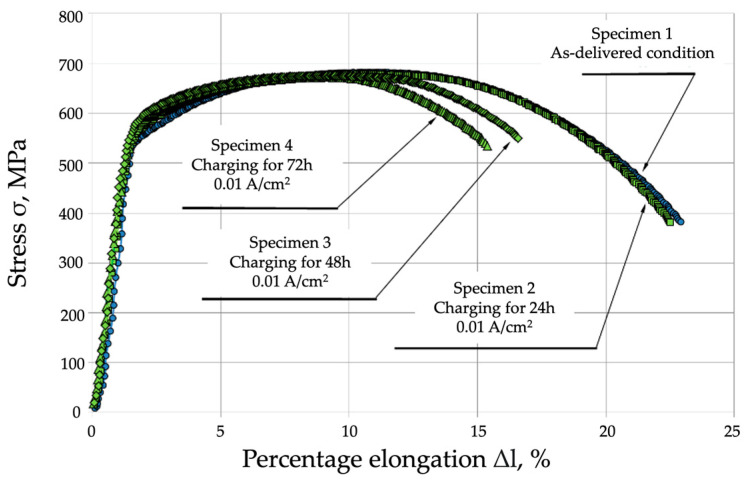
Comparison of the static tensile strength of P91 steel in the as-delivered condition and following hydrogen charging without preloading.

The results of the static tensile strength tests on P91 steel in the as-delivered condition enabled the determination of the basic mechanical properties of the material tested. The values obtained were later used in further testing phases. Table 1 and Figure 5 compare the results obtained for the specimens tested in the as-delivered condition, and those tested following hydrogen charging without load.

**Figure 6:**
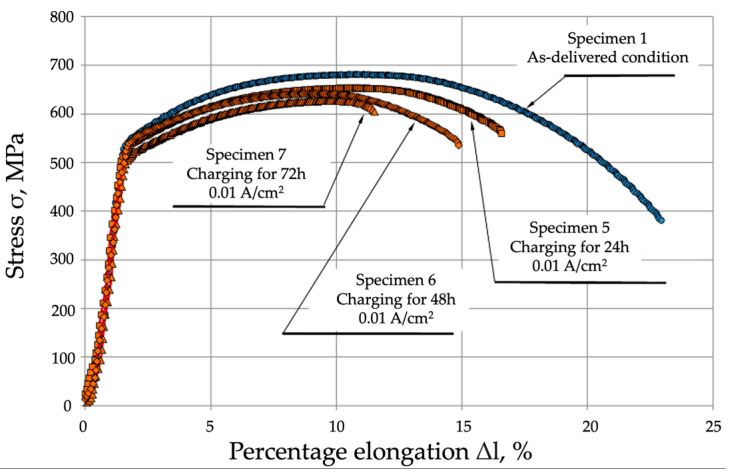
Comparison of the static tensile strength of P91 steel in the as-delivered condition and following hydrogen charging under preload.

Table 2 and Figure 6 compare the results obtained for the specimens tested in the as-delivered condition and those tested following hydrogen charging under load.

**Figure 7:**
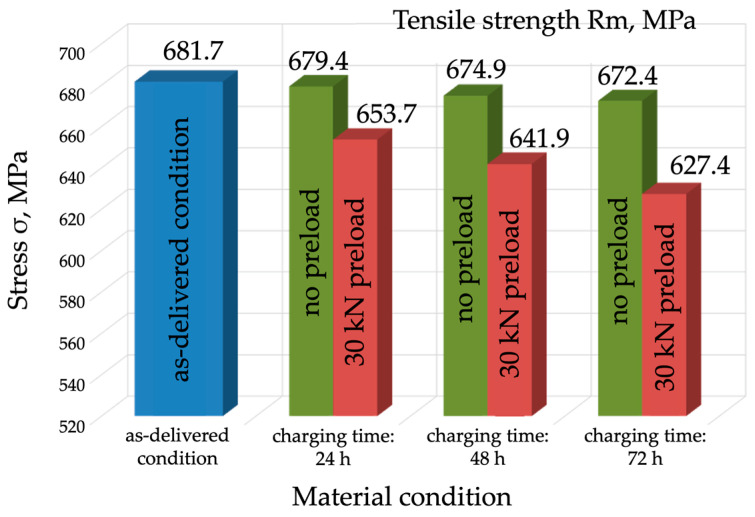
Tensile strength comparison for the different variants of the hydrogen charging test.

**Figure 8:**
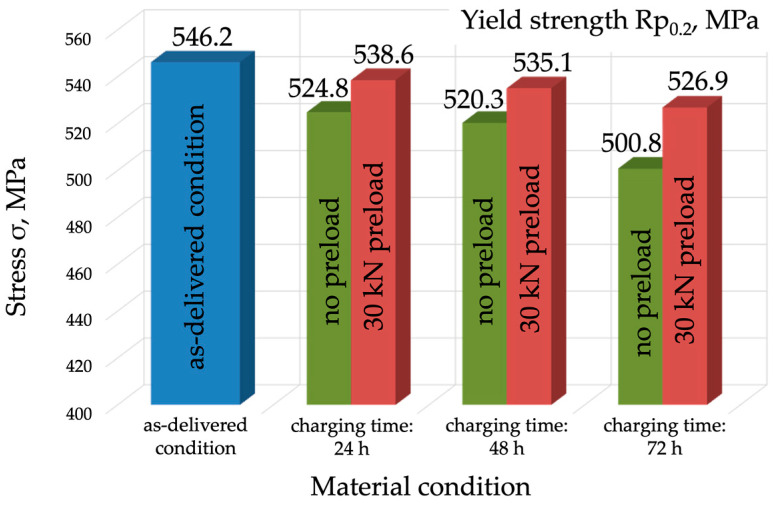
Yield strength comparison for the specimens in the as-delivered condition following hydrogen charging without load, and following hydrogen charging under a load of 30 kN.

**Figure 9:**
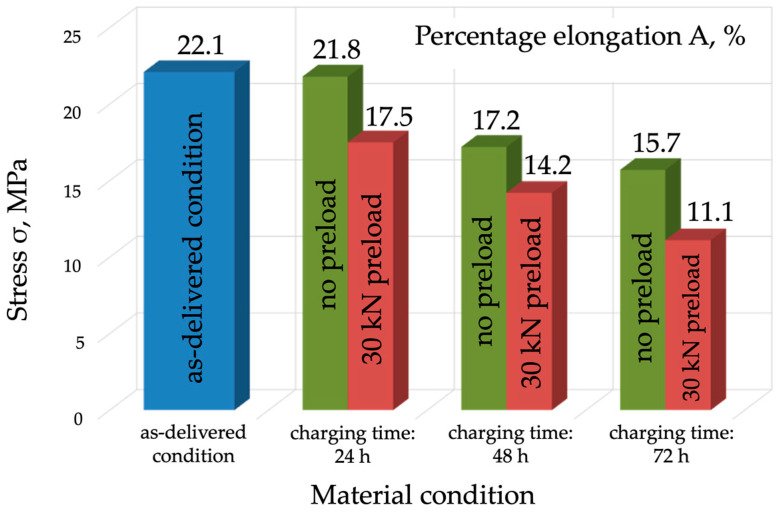
Percentage elongation comparison for the specimens in the as-delivered condition, following hydrogen charging without load, and following hydrogen charging under a load of 30 kN.

**Figure 10:**
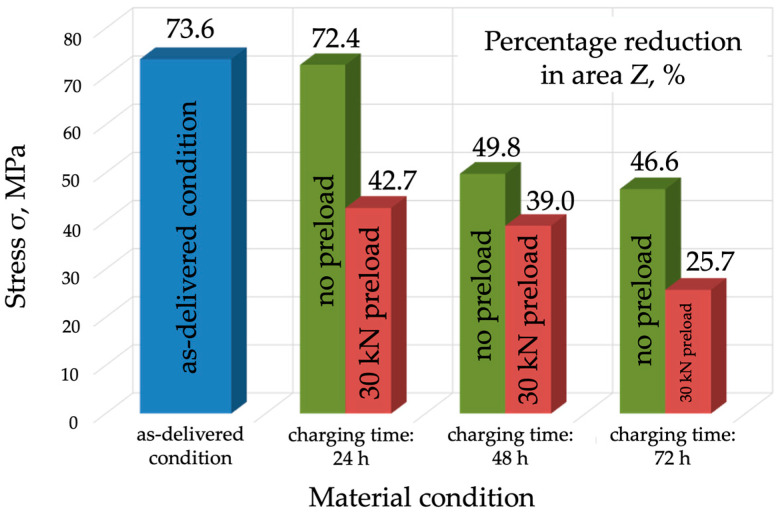
Percentage reduction in area comparison for the specimens in the as-delivered condition following hydrogen charging without load, and following hydrogen charging under a load of 30 kN.

The analysis of the results obtained during the tests (for R_m_, R_e_, A_5_, and Z) clearly indicates that hydrogen causes a reduction in both strength and plasticity-related properties (Figure 7, Figure 8, Figure 9 and Figure 10). A slight reduction in the material’s strength was identified even after 24 h of exposure to hydrogen. In the case of the tests without preloading, the strength of the material decreased as the charging time increased and fell to 672 MPa after 72 h, which means a decrease of 10 MPa (Figure 7). It should be concluded that, under these conditions, with strength as the criterion, P91 steel can be deemed resistant to hydrogen-containing environments. Much worse strength results were obtained for the specimens that were hydrogen-charged under constant load and then subjected to the tensile strength tests.

As early as after 24 h, the material’s strength decreased to 654 MPa, and after 72 h it decreased to 627 MPa. This confirms that hydrogen affects the mechanical properties of P91 steel, especially during service and operation.

As for the yield strength (R_p0.2_), which is 546 MPa in the as-delivered condition, it decreased as the charging time increased, falling to 501 MPa after 72 h of charging without load (Figure 8). The analysis of the results obtained for the specimens charged under load showed a reduction in yield strength compared to the value obtained for the material in the as-delivered condition (by 20 MPa), but an increase in yield strength compared to the specimens charged without load. The difference in R_p0.2_ between the specimens charged without load and under load was 13.8 MPa for the charging time of 24 h, and 26.1 MPa for the charging time of 72 h (Figure 8).

The evaluation of plastic properties (A, Z) showed a significant decrease in the ductility of the specimens exposed to hydrogen in comparison with the specimens in the as-delivered condition (Figure 8 and Figure 9). In particular, a considerable deterioration in plastic properties was observed for the specimens that were hydrogen-charged under load. The specimens charged without load for 72 h showed a 6% decrease in percentage elongation A, which is 29% less compared to the as-delivered condition (Figure 9). The elongation A was 11%, which was a 50% decrease compared to the as-delivered condition.

The analysis of the results for reduction in area (Z) of P91 steel confirmed a decrease in ductility in the specimens exposed to hydrogen, especially those charged under load. The percentage reduction in area (Z) for the specimens charged without load for 72 h was 46.6%, which was a 37% reduction compared to the as-delivered condition. For the specimens charged under load for 72 h, the reduction in area was 25.7%, which was a 65% decrease compared to the as-delivered condition (Figure 10).

An additional static tensile test was conducted on the material under load of 30 kN for 110 h. The results demonstrate a significant degradation of the material’s plastic properties. The elongation at fracture (A) was measured at 3.4%, while the reduction in area (Z) was recorded at 1.6%. These findings indicate a substantial negative impact of hydrogen on the plastic properties of P91 steel, particularly in operating conditions characterized by sustained load and a hydrogenation environment. A rapid increase in embrittlement can lead to the swift and uncontrolled failure of the entire structure.

**Figure 11:**
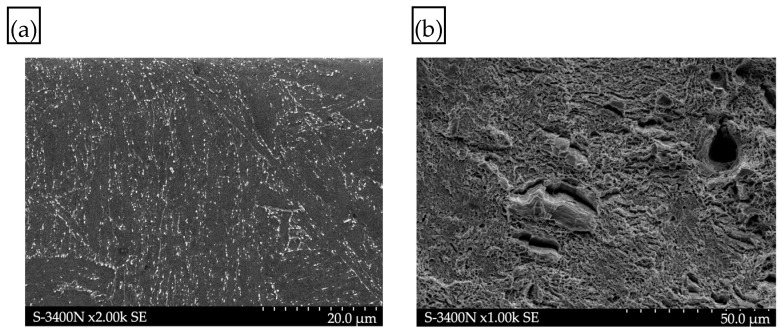
Structure of P91 steel in the as-delivered condition: (**a**) martensitic structure on a flat cross-section; (**b**) mixed brittle–ductile fracture surface following the static tensile strength test.

**Figure 12:**
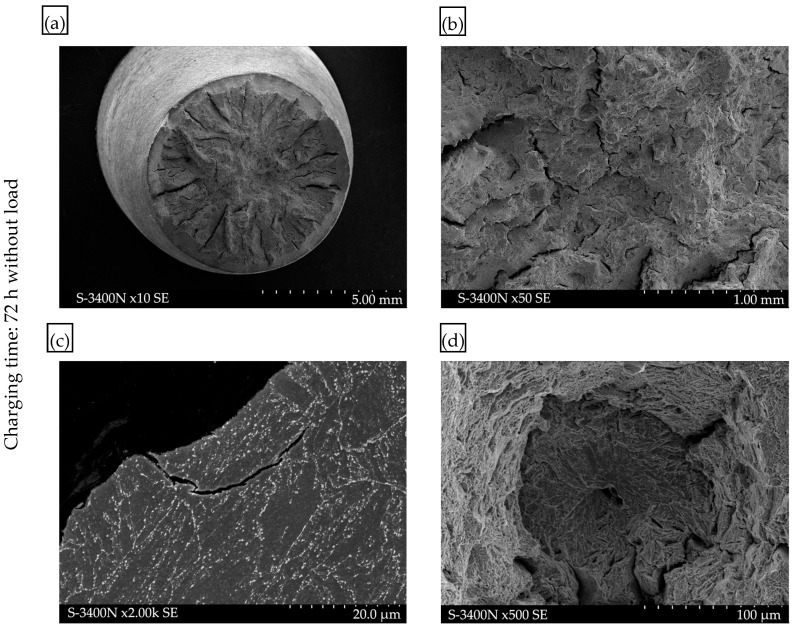
Fracture surface after static tensile testing following hydrogen charging without load for 72 h: (**a**) overall view of the fracture surface; (**b**) mixed ductile-brittle fracture structure with visible cleavage fracture; (**c**) intergranular cracks in the fracture zone; (**d**) fracture surface with a visible “fish-eye” hydrogen crack.

**Figure 13:**
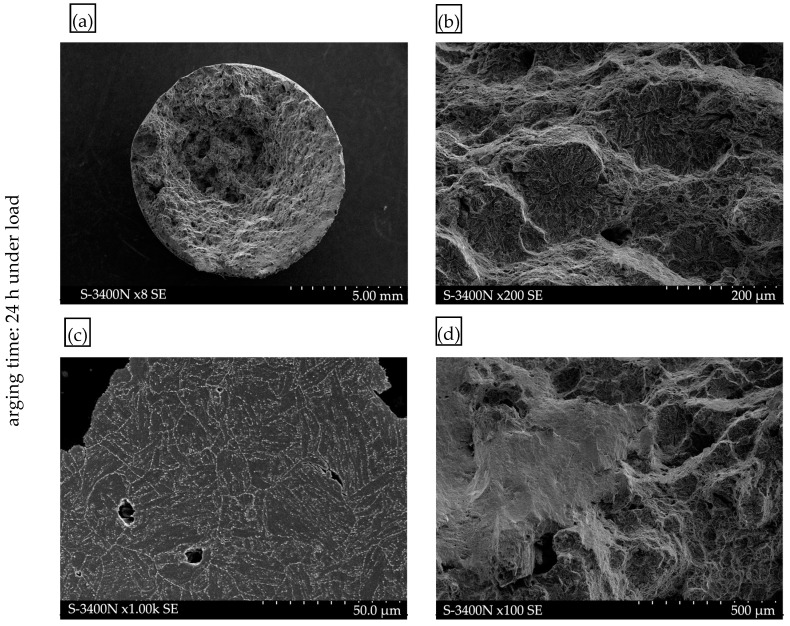
Fracture surface after static tensile testing following hydrogen charging with load for 72 h: (**a**) overall view of the fracture surface; (**b**) fracture surface with visible “fish-eye” overlap cracks; (**c**) microstructure in the fracture zone (flat cross-section), revealing voids and blisters associated with hydrogen presence, with microcracks along grain boundaries; (**d**) smooth surface of the hydrogen crack on the fracture surface.

The mechanical tests were complemented by fractographic examinations of the fracture surfaces. Such images clearly show surface topography. Examples of fracture surfaces for the material in the as-delivered condition are shown in Figure 11, whereas Figure 12 and Figure 13 show fracture surfaces of specimens tested following hydrogen charging without load and under load, respectively.

After the static tensile test, the sample exhibited a ductile fracture with visible microcracks and micropores (Figure 11b). Examination of the sample’s side surface revealed no microcracks or other discontinuities, indicating good mechanical properties and a lack of susceptibility to microcrack formation, which could potentially lead to structural failure.

Analysis of the results of the static tensile test of P91 steel samples without load did not reveal any significant changes in the mechanical properties of the material after 24 h of sample exposure in the solution environment. The material strength decreased by 10 MPa (Figure 8), and the yield strength decreased by 21 MPa. Significant decreases in area reduction and elongation were observed after 48 h and 76 h of exposure, while the strength of the material remained relatively unchanged. These results suggest that P91 steel exhibits significant resistance to hydrogen embrittlement up to 24 h, however, prolonged exposure to hydrogen leads to increased embrittlement. The yield strength (R_p0.2_) decreased from 546 MPa to 501 MPa after 72 h of exposure to the hydrogenated solution without load. Samples hydrogenated without load for 72 h exhibited a 6% decrease in elongation (A), representing a 29% reduction compared to the initial state. The percentage reduction in area (Z) for the sample hydrogenated without load for 72 h was 46.6%, indicating a 37% decrease compared to the initial state.

Analysis of fractures and microstructure on flat longitudinal metallographic sections reveals the presence of typical HELP phenomenon transcrystalline microcracks, attributed to a reduction in the strength of the material’s micro-regions (Figure 12a,b). Additionally, cracks are visible along grain boundaries. This is a phenomenon associated with hydrogen accumulation in chromium carbide precipitates and the grain boundary area (HEDE) (Figure 12c). The fracture surface also exhibits several defects described as “fish eyes” (Figure 12d), characteristic of the AIDE phenomenon and likely caused by hydrogen accumulation at the interface between metallic inclusions and the matrix. The obtained data confirm that P91 steel exhibits susceptibility to hydrogen embrittlement, consistent with the observed decline in mechanical properties.

Fractographic analysis of the surfaces indicates a mixed ductile-brittle fracture mode in all cases. A detailed examination of the fracture surfaces of samples subjected to hydrogen charging under load revealed numerous effects of structural degradation due to hydrogen interaction (Figure 13). Numerous “fish-eye” cracks were observed on the fracture surface of the sample after 72 h of exposure to a hydrogen-charging environment under a constant load (Figure 13b). These cracks were so numerous that they overlapped, leading to the formation of microcracks and even larger cracks perpendicular to the tensile direction. It is a typical effect of hydrogen embrittlement, leading to a loss of material cohesion. Example observations of these microcracks are shown in Figure 13c, where the smooth surface of the microcrack, caused by hydrogen interaction, is visible. This phenomenon is described in the literature as HELP and AIDE [25,26,28]. Additionally, numerous voids and cracks caused by the presence of hydrogen were observed in the structure. An example of the observed structure from the fracture area with visible hydrogen voids and microcracks along the grain boundaries is shown in Figure 13c.

Further metallographic analysis of cross-sections transverse to the tensile direction revealed numerous cracks on the sample surface, which is associated with the interaction of hydrogen according to the mechanism described as HEDE [25,27].

In conclusion, it can be positively stated that a hydrogen-containing environment has a very negative effect on the mechanical properties of P91 steel. With long hydrogen exposure times under load, brittleness sharply increases, which is very dangerous from the operational point of view. An important factor affecting the material’s behavior in a corrosive, hydrogen containing environment is the additional stress present during hydrogen charging. This is particularly important because these conditions are the closest to the actual operating conditions of most industrial facilities. In this study, a preload of 70% of R_p0.2_ was applied during the hydrogen charging process, which simulated extremely dangerous conditions.

## 4. Conclusions

Based on the tests conducted and the analysis of their results, the following conclusions were formulated:

- Mechanical properties are affected by the environment and the way in which the mechanical properties of P91 steel are affected by the environment and the way in which the material is exposed to hydrogen. Exposure without load for 72 h caused a slight decrease in the material’s strength, by approx. 10 MPa, however, under a load of 0.7 Re, the material’s strength decreased by 60 MPa (approx. 10%). A different mode was observed for the change in yield strength (Re), which was 546 MPa in the as-delivered condition. Following exposure to a hydrogen environment for 72 h without load, the material’s yield strength fell to 501 MPa, however, under a load of 30 kN, it decreased to 527 MPa.
- The plastic properties of P91 steel deteriorated significantly as the hydrogen charging time increased. In the as-delivered condition, elongation A5 was 22.1%, and reduction in area Z was 76.6. Following hydrogen charging under load, A5 decreased to 11.1% (a decrease of almost 50%), while reduction in area Z fell to 25.7% (a decrease of 75%).
- The adverse effect of hydrogen on the mechanical properties, including plastic properties, of P91 steel was confirmed by the examinations of the fracture surfaces following the static tensile strength tests. In the specimens exposed to hydrogen, the proportion of brittle fracture was higher and the so-called “fish-eyes” were observed, which indicated hydrogen trapping in the structure.
- The operation of P91 steel facilities in a corrosive, hydrogen containing environment significantly accelerates its structural degradation due to the adverse effect of hydrogen, especially if the facility operates under continuous stress. This translates directly into increased brittleness, which contributes to uncontrolled failure. This is particularly dangerous in the context of the operation of industrial facilities.

## Figures and Tables

**Table 1:** Mechanical properties of P91 steel specimens subjected to static tensile strength tests in the as-delivered condition and following hydrogen charging without preloading.

Specimen ID Charging Time	Tensile Strength Rm, MPa	Yield Strength R_p0.2_, MPa	Percentage Elongation A, %	Percentage Reduction in Area Z, %
Specimen 1—As-delivered condition	682 ± 2.45	546 ± 1.96	22.1	73.6
Specimen 2—Charging for 24 h	679 ± 3.27	525 ± 2.53	21.8	72.4
Specimen 3—Charging for 48 h	674 ± 4.9	520 ± 3.78	18.2	49.8
Specimen 4—Charging for 72 h	672 ± 6.53	501 ± 4.12	15.7	46.6

**Table 2:** Mechanical properties of P91 steel specimens subjected to static tensile strength tests in the as-delivered condition and following hydrogen charging under load.

Specimen ID Charging Time	Tensile Strength Rm, MPa	Yield Strength R_p0.2_, MPa	Percentage Elongation A, %	Percentage Reduction in Area Z, %
Specimen 1—As-delivered condition	682 ± 3.26	546 ± 2.61	22.1	73.6
Specimen 2—Charging for 24 h	654 ± 5.71	539 ± 4.71	17.5	43.7
Specimen 3—Charging for 48 h	642 ± 7.34	535 ± 6.11	14.2	39.0
Specimen 4—Charging for 72 h	627 ± 8.98	527 ± 7.55	11.1	25.7

## Data Availability

The data presented in this study are available on request from the corresponding author.
